# Influence of Sampling on the Determination of Warfarin and Warfarin Alcohols in Oral Fluid

**DOI:** 10.1371/journal.pone.0114430

**Published:** 2014-12-05

**Authors:** Tommaso Lomonaco, Silvia Ghimenti, Isabella Piga, Denise Biagini, Massimo Onor, Roger Fuoco, Fabio Di Francesco

**Affiliations:** 1 Department of Chemistry and Industrial Chemistry, University of Pisa, Pisa, Italy; 2 Institute of Chemistry of Organometallic Compounds, CNR, Pisa, Italy; 3 Institute of Clinical Physiology, CNR, Pisa, Italy; Ehime University Graduate School of Medicine, Japan

## Abstract

**Background and Objective:**

The determination of warfarin, RS/SR- and RR/SS-warfarin alcohols in oral fluid may offer additional information to the INR assay. This study aimed to establish an optimized sampling technique providing the best correlation between the oral fluid and the unbound plasma concentrations of these compounds.

**Materials and Methods:**

Samples of non-stimulated and stimulated oral fluid, and blood were collected from 14 patients undergoing warfarin therapy. After acidification, analytes were extracted with a dichloromethane/hexane mixture and determined by HPLC with fluorescence detection. Plasma samples were also ultrafiltered for the determination of the unbound fraction. The chromatographic separation was carried out in isocratic conditions with a phosphate buffer/methanol mobile phase on a C-18 reversed-phase column. The absence of interfering compounds was verified by HPLC-ESI-Q-TOF.

**Results:**

Stimulation generally increased the oral fluid pH to values close to blood pH in about 6 minutes. The concentration of warfarin and RS/SR-warfarin alcohols in oral fluid followed the same trend, whereas the concentration of RR/SS-warfarin alcohols was not affected. Six minute stimulation with chewing gum followed by collection with a polyester swab was the best sampling procedure, with a good repeatability (RSD <10%) and relatively low inter-subject variability (RSD  = 30%) of the oral fluid to plasma ratio. This procedure provided strong correlations between the measured oral fluid and unbound plasma concentration of warfarin (r  =  0.92, p <0.001) and RS/SR-warfarin alcohols (r  =  0.84, p <0.001), as well as between stimulated oral fluid and total plasma concentration of warfarin (r  =  0.78, p <0.001) and RS/SR-warfarin alcohols (r  =  0.81, p <0.001).

**Conclusion:**

The very good correlation between oral fluid and unbound plasma concentration of warfarin and RS/SR-warfarin alcohols suggests that oral fluid analysis could provide clinically useful information for the monitoring of anticoagulant therapy, complementary to the INR assay.

## Introduction

**Figure 1:**
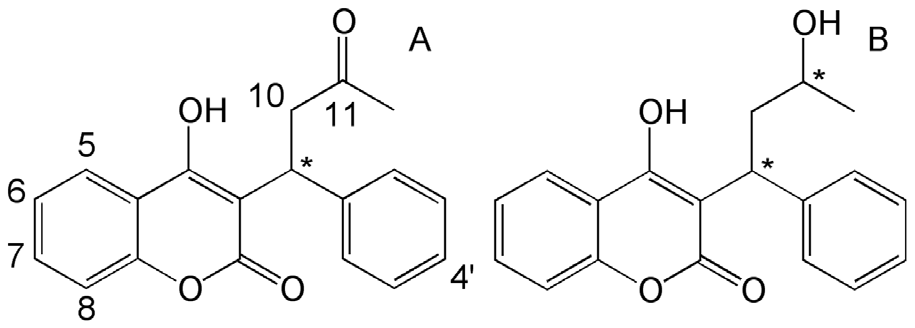
Structures of WAR (A) and WAROHs (B) * asymmetric center.

Warfarin [3-(α-acetonylbenzyl)-4-hydroxycoumarin] (WAR, **Figure 1A**) is the most common anticoagulant drug prescribed for the treatment of many diseases (e.g. atrial fibrillation and pulmonary embolism) [1]. WAR has a narrow therapeutic range (2 <INR <3) [2] and is prone to interferences (from other drugs and food) that may enhance or reduce the anticoagulation effect [3], [4]. WAR is metabolized in the liver by the cytochrome (CYP) P450 to inactive hydroxylated metabolites (OH-WAR) (major pathway) and by ketone reductases to RS/SR-warfarin alcohols and RR/SS-warfarin alcohols (WAROHs, **Figure 1B**) [5]. WAROHs have a limited anticoagulant activity with a half maximal inhibitor concentration (IC_50_) of 12.5 µM (IC_50_ of WAR is 2.2 µM) [6].

The anticoagulant activity of WAROHs may be an additional variable involved in the anticoagulant mechanism of WAR [7], but there is little pharmacological data concerning these metabolites.

Evaluation of the anticoagulation level by the international normalized ratio (INR) is the primary assay used to monitor WAR therapy [8]. When a patient begins WAR therapy, blood sampling and INR monitoring should be performed on a daily basis until the value falls within the therapeutic range. Then the INR should be checked two or three times a week for one to two weeks, then once every two weeks or a month provided that the values permanently lie within the correct therapeutic range [9], [10].

The main problems with this approach are the large variability in the subjects' responses to treatment and the delayed anticoagulant effect of WAR (≈72 hours) [9], which risk the onset of hemorrhagic events. The social and economic costs related to the frequent access of patients to anticoagulation centers are further drawbacks to this therapy.

The determination of WAR and its active metabolites (WAROHs) in oral fluid samples (OF) could offer additional information to the INR assay, because the OF concentration of WAR is expected to mirror the concentration of unbound WAR in plasma (the fraction determining the pharmacological efficacy [11]) and could anticipate the INR variations. Finally, compared to blood and its derivatives, OF is easily collected, has a low risk of infection, and does not need specially-trained personnel to administer it [12]. For example, this approach was successfully applied by Knott *et al* to evaluate the interaction between phenytoin and valproate in patients undergoing epilepsy therapy [13].

Ghimenti *et al*, suggested the existence of a correlation between OF concentration of WAR and INR in patients with OF pH ≥ 7.2 (r  =  0.84, p <0.001), but the limited number of patients with this pH value did not lead to a statistically firm conclusion [14]. Nevertheless, this result confirmed that OF pH plays an important role in the diffusion process of WAR from blood to oral fluid across the salivary gland membrane. OF concentrations of basic and acidic drugs (such as WAR, pK_a_  =  5.15 ± 0.04 at T  =  25°C) [15]) are largely dependent on variations of the OF pH [16] and an optimized method for stimulated OF sample collection is required [17]. In fact, the stimulated secretion of OF increases the concentration of bicarbonate ions, which determine higher pH values and a moderate buffering effect [18]. Thus, the oral fluid/plasma concentration ratio (OF/P) should be more constant in stimulated than in non-stimulated OF [16].

There are several methods for collecting non-stimulated (draining, spitting, suction and adsorption into swab) and stimulated (with chemical or masticatory stimulus) OF samples [19]. The OF secretion can be stimulated by applying a few drops of citric acid (0.1–0.2 mol/L) directly onto the tongue, or chewing paraffin wax, parafilm, rubber bands or chewing gum. After stimulation, the OF can be spat out, suctioned or absorbed [19]. For hydrophilic drugs, different salivary flow rates obtained with different sampling methods may influence the measured drug concentration in OF, as a more effective stimulation leads to a larger dilution of the sample [20], [21].

Based on this background information, we developed a reliable analytical method for the determination of WAR and both diastereoisomers of WAROHs in OF samples by high performance liquid chromatography with fluorescence detection (HPLC-FLD), which included cleanup and pre-concentration steps. Instrumentation needed for HPLC-FLD is typically available in the clinical laboratories, and so the use of this technique fosters the transfer of research results to the clinical routine. The method was then applied to samples from patients undergoing WAR therapy. We compared stimulated and non-stimulated OF samples in order to find the right sampling conditions to obtain the best correlation between OF and plasma concentrations of unbound WAR, RR/SS-warfarin and RS/SR-warfarin alcohols.

## Materials and Methods

### Statement of ethics

Ethical permission was obtained from the Ethics Committee of the Azienda Ospedaliero-Universitaria Pisana. Patients undergoing WAR therapy and nominally healthy subjects who volunteered to join the project gave written informed consent prior to their participation.

### Study subjects

Fourteen patients (9 males, 5 females) undergoing WAR therapy were recruited. The enrolled subjects were treated for atrial fibrillation (AF, 60%), deep vein thrombosis (DVT, 10%) or were mechanical or biological heart valve bearers (MHV, 30%). Their average age was 67 ± 14 years (range, 38–87 years) and the average WAR dose was 25.50 ± 15.50 mg/week (range, 1.25–48.75 mg/week). INR values varied from 1.8 to 3.7, with an average value of 2.6 ± 0.6. The Mann-Whitney test did not highlight statistically significant gender differences (p <0.05) for any of the above parameters. Twenty nominally healthy subjects who were not taking any drug also contributed to the project by providing control OF samples.

### Chemicals

Racemic WAR, i.e. 3-(α-acetonylbenzyl)-4-hydroxycoumarin sodium salt (purity ≥ 98.0%), sodium borohydride (purity ≥ 98.0%), sodium hydroxide solution, sulfuric acid (purity ≥ 99.9%), phosphoric acid 85 wt% in H_2_O (purity ≥ 99.9%), ethanol (purity ≥ 99.5%) and deuterated methanol were purchased from Sigma Aldrich (Italy). Sodium phosphate monobasic (purity ≥ 99.0%), potassium phosphate dibasic (purity ≥ 99.0%), dichloromethane (purity ≥ 99.5%), hexane (purity ≥ 95.0%), formic acid (purity ≥ 99.5%), acetonitrile and methanol were purchased from Sigma Aldrich at HPLC grade. The certified buffer solutions at pH  =  3, 4, 5, 6 and 7 were purchased from Sigma Aldrich (Italy) to calibrate the pH-meter daily.

WAROHs were obtained in our laboratory by quantitatively reducing a racemic WAR standard solution with sodium borohydride [22], [23]. The HPLC-FLD separation of the reduced solution revealed that the reaction with sodium borohydride was completed and WAROHs were generated. The product of reduction was confirmed by comparing the ^1^H NMR and ^13^C NMR spectra of WAR and WAROHs [23]. The concentration of each diastereoisomer (i.e. RR-SS and RS-SR) in the stock solution of WAROHs was estimated by analyzing HPLC-UV-Vis signals. Assuming the same instrumental sensitivity for the two compounds, which is a reasonable hypothesis for such similar molecules, and given that the ratio of the areas of the two signals was equal to 1, the solution was considered to contain equimolar amounts of RR-SS and RS-SR. The absence of additional peaks made us conclude that the synthetized compound did not show impurities. HPLC grade water was produced by a Milli-Q Reagent Water System (Millipore, USA).

### Instrumentation

HPLC-FLD analysis was carried out using a Jasco HPLC system equipped with an autosampler (AS 2055), a quaternary low-pressure gradient pump (PU 2089), a fluorescence detector (FP 2020), and an ultraviolet detector (UV 2070). The column temperature was controlled by a thermostat (HT 3000, ClinLab). The HPLC-FLD system was controlled using ChromNAV software from Jasco.

The chromatographic separation of WAR and WAROHs was carried out on a C-18 reversed-phase column Poroshell EC-C18 (Agilent, 100×4.6 mm, 2.7 µm) connected to a guard column TC-C18 (Agilent, 12.5 × 4.6 mm, 5 µm).

A few selected OF samples were also analyzed by an Agilent HPLC-ESI-Q-TOF system to compare the analytical methods. The LC-MS analyses were carried out using a 1200 Infinity HPLC coupled to a 6530 Accurate-Mass Quadrupole Time-Of-Flight (Q-TOF) mass spectrometer with a Jet Stream ESI interface (Agilent Technologies, USA). The HPLC system was controlled by MassHunter software from Agilent. Chromatographic separation of WAR and WAROHs was carried out on a C-18 reversed-phase column Zorbax Extend-C18 (Agilent, 50×2.1 mm, 1.8 µm) connected to a guard column Eclipse Plus (Agilent, 12.5×4.6 mm, 5 µm).

A pH 1100 bench pH-meter (Eutech Instruments, Singapore) was used to check the pH of the mobile phase.

A centrifuge Centrifugette 4206 (ALC, Italy) and a Centrifuge 5804 R equipped with an A-4-44 swinging bucket rotor (Eppendorf, Italy) were used for sample centrifugation.

All data were analyzed using GraphPad Prism v.6 from GraphPad Software Inc.

### Experimental conditions

HPLC-FLD separation of WAR and WAROHs in extracted OF and plasma samples as well as in centrifuged plasma samples was carried out in isocratic mode with a mobile phase consisting of 30% methanol and 70% phosphate buffer 25 mM at pH  =  7, at a flow rate of 0.7 mL/min, injection volume 25 µL. Fluorescence detection was performed at excitation and emission wavelengths of 310 and 390 nm, respectively. The total HPLC-FLD run time was 26 minutes.

HPLC-ESI-Q-TOF determination of WAR and WAROHs was carried out in isocratic mode with a mobile phase of 35% acetonitrile and 65% water containing 1% formic acid at a flow rate of 0.25 mL/min at 25°C, injection volume 4 µL. The ESI operating conditions were: drying gas (N_2_, purity>98%): 350°C at 10 L/min; capillary potential 4.5 KV; nebulizer gas 35 psig; sheath gas (N_2_, purity>98%): 375°C at 11 L/min. The fragmentor was kept at 100 V and the collision energy (CID) for the MS/MS experiments was 20 V. The collision gas was nitrogen (purity 99.999%). High-resolution MS and MS/MS spectra were achieved in positive mode in the range 100-350 m/z. The protonated molecular ions [M+H]^+^ with m/z 311.1 and 309.1 were monitored for WAROHs and WAR identification, respectively. The total HPLC-ESI-Q-TOF run time was 10 minutes. The linear regression of the peak area versus concentrations was fitted over the concentration range of 1–1000 ng/mL for both WAR and WAROHs. The seven-point calibration curves (n  =  3 at each concentration) were evaluated by the Deming regression analysis, and the best-fit models were (the slope values are reported with the corresponding standard deviation): y  =  (540 ± 10) x, (R^2^ =  0.999) for RR/SS-warfarin alcohols, y  =  (1110 ± 10) x, (R^2^ =  0.998) for RS/SR-warfarin alcohols and y  =  (1740 ± 10) x, (R^2^ =  0.999) for WAR.

Five aliquots of a blank oral fluid sample spiked with 0.5 ng/mL of both WAR and WAROHs were treated according to the optimized procedure and then analyzed by HPLC-ESI-Q-TOF. The LOD and LOQ values were calculated, in accordance with IUPAC guidelines [24], as three and ten times the standard deviation (s_b_) of the low level “spiked blank”, and resulted in: 0.7 and 2 ng/mL for both WAR and RS/SR-warfarin alcohols, and 0.3 and 1 ng/mL for RR/SS-warfarin alcohols.

### Standard solutions and quality control samples

A phosphate buffer solution (PBS) (1 M, pH  =  7.0) was prepared by dissolving 17.90 g of sodium phosphate monobasic and 49.10 g of potassium phosphate dibasic in water. This solution was diluted to 25 mM to obtain the PBS used for the HPLC-FLD analyses and for the preparation of standard solutions.

Stock solutions of WAR (970 µg/mL) and WAROHs (1040 µg/mL) were prepared by dissolving weighed amounts of the compounds in water and methanol respectively. These solutions were used to prepare another stock solution containing WAR (950 ng/mL) and WAROHs (1060 ng/mL) in PBS 25 mM, which was further diluted with PBS to obtain standard working solutions of WAR and WAROHs at 1, 2, 5, 10, 20, 100 and 200 ng/mL. The standard solutions, protected from light and stored at 4°C, were stable for more than two months [23].

Pooled patient oral fluid samples (PPOFSs) were obtained by pooling samples from 14 patients undergoing WAR therapy, whereas pooled control oral fluid samples (PCOFSs) were obtained from 20 volunteers not taking WAR. Pooled samples allow to bring into experiments the high variability between different individuals, maximizing the inclusion of potentially interfering compounds. For this reason, they allow to perform a much more severe test compared to the use of single patients' samples with a limited experimental effort.

Aliquots of a PCOFS were spiked with known amounts of WAR and WAROHs to obtain pooled standard oral fluid samples (PSOFSs) at different concentration levels. PSOFSs were stored at −80°C, and were checked for stability over a period of two months and after thaw-freeze cycles.

### Octanol-Water partition coefficient (K_ow_) and dissociation constant of RR/SS- and RS/SR-warfarin alcohols

The LogK_ow_ value at pH  =  7 of both diastereoisomers of WAROHs was determined using the standardized OECD guidelines [25]. Briefly, a standard working solution of WAROHs (1000 ng/mL, C1) was prepared in triplicate in the PBS buffer (25 mM at pH  =  7) and then mechanically stirred with an equal volume (4 mL) of 1-octanol at 25°C for 24 hours. The phase separation was obtained at room temperature by centrifuging at 5000 rpm for 5 min. The concentration of RR/SS-warfarin alcohols (C2) and RS/SR-warfarin alcohols (C3) in the aqueous phase was determined by the HPLC-FLD method. The concentration in the organic phase was calculated by subtracting C2 and C3 from C1. LogK_ow_ at pH  =  7 is expressed as the ratio of the compound concentration in the organic phase to that in the aqueous phase.

The dissociation constant (pK_a_) value of RR/SS- and RS/SR-warfarin alcohols was measured using a modified version of the method proposed by Manderscheid M and Eichinger T [26]. In brief, a standard working solution of WAROHs (1060 ng/mL) was prepared in a PBS buffer (25 mM at different pH value) and then analyzed in triplicate by HPLC-FLD. This approach is based on the different chromatographic behaviour of dissociated and non-dissociated forms of each compound at different pH values of the mobile phase. The pH was lowered in steps of 1 unit and ranged from 7.0 to 3.0. To reach the required pH, different aliquots of H_3_PO_4_ (85 wt% in H_2_O) were added to the mobile phase. The resulting pH was further checked by a pH-meter.

The capacity factor to pH variation ratio (Δ*k’*/ΔpH) of both diastereoisomers was determined with different contents of organic modifiers (R_wt%_: 40, 50 and 60) in the methanol-PBS mobile phase. The cosolvent dissociation constant (p_s_K_a_) value was calculated by equation 1 [27]:
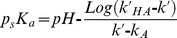
(1)where *k’*_HA_ and *k’*_A_ are the capacity factors of non-dissociate and dissociate forms, respectively. The pH and *k’* values were defined as the corresponding point with a minimum slope in the Δ*k’*/ΔpH plot. From the traditional plot of p_s_K_a_ *vs* R_wt%_, the best aqueous pK_a_ value of RR/SS- and RS/SR-warfarin alcohols was obtained by extrapolating at zero organic content.

### Sample collection

#### Oral fluid sample collection

The patients took WAR at about 6 PM and did not take any food or beverages within 1 h prior to OF collection. OF samples were collected in a quiet room between 7 and 10 AM just after a blood sample had been taken using a biocompatible roll-shaped polyester Salivette swab (Sarstedt, Germany).

Non-stimulated OF samples were collected by asking the subjects to place the swab in the mouth, between the gum and cheek, and to keep it steady for 10 min (no chewing or movements, procedure 1). To obtain stimulated OF samples two procedures were tested on different days. The subjects rolled a swab with their tongue for 2 min (procedure 2) or chewed sugar-free chewing gum for 6 min (procedure 3). In both sampling procedures, after the stimulation step, the OF was immediately collected at five different times (t  =  2, t  =  4, t  =  6, t  =  8 and t  =  10 min) by rolling another synthetic swab for 2 min. The amount of the absorbed OF sample was calculated according to weight differences before and after sampling. From these values, the OF flow rate in grams per minute (almost equivalent to milliliters per minute, [28]) was calculated. After OF sampling, the pH value was measured by two independent observers using a narrow range (resolution of 0.3 pH units) pH paper strip (Pehanon, Macherey Nagel). Oral fluid collection and pH measurements were always completed in less than 30 minutes.

The OF was recovered by centrifugation of the swabs at 3000 rpm for 5 min at room temperature. Finally, the sample was frozen at −80°C until assay.

#### Blood sample collection

The plasma samples were obtained by the usual standardized procedure used in clinics: human whole blood samples were collected into vacuum tubes containing 109 mM (3.2%) sodium citrate (Vacutest Kima, Italy) and immediately centrifuged at 3000 rpm for 10 min at room temperature to obtain platelet-poor plasma. The plasma sample was stored at −80°C until assay.

### Sample preparation

#### Oral fluid sample preparation

The aim of the sample preparation was to remove the endogenous components (e.g. proteins, salts, lipids) that might interfere with the chromatographic analysis. An aliquot of OF (1 mL) was added with 2 mL of H_2_SO_4_ 0.5 M, 1 mL of ethanol, and 4 mL of a mixture of dichloromethane/hexane (1:5 v/v). The resulting mixture was vortex-mixed for 30 sec and then centrifuged at 5000 rpm for 5 min at room temperature. The organic phase was recovered, evaporated under nitrogen and reconstituted in 0.25 mL of PBS 25 mM at pH  =  7. An aliquot (25 µL) of this solution was injected into the HPLC-FLD system for the determination of WAR and RR/SS- and RS/SR-warfarin alcohols.

#### Plasma sample preparation

The procedures to determine WAR and WAROHs in plasma samples are described in [23]. To determine the total content of WAR and RR/SS- and RS/SR-warfarin alcohols, an aliquot of plasma (0.5 mL) was added with 2 mL of H_2_SO_4_ 0.5 M, 0.5 mL of ethanol, and 4 mL of a mixture of dichloromethane/hexane (1∶5 v/v). The resulting mixture was vortex-mixed for 30 sec, and then centrifuged at 5000 rpm for 5 min at room temperature. The organic phase was recovered, evaporated under a mild nitrogen flux, and reconstituted in 1 mL of PBS 25 mM at pH  =  7. An aliquot (15 µL) of solution was then injected into the HPLC-FLD system.

The seven-point calibration curves (n  =  3 at each concentration) in the range 100–3000 ng/mL were evaluated by the Deming regression analysis, and the best-fit models were (the slope values are reported with the corresponding standard deviation): y  =  (20300 ± 100) x, (R^2^ =  0.999) for RR/SS-warfarin alcohols, y  =  (7700 ± 100) x, (R^2^ =  0.999) for WAR and y  =  (12000 ± 200) x, (R^2^ =  0.999) for RS/SR-warfarin alcohols.

Inter- and intra-day precision and recovery were determined for spiked plasma samples at 200, 1000 and 2000 ng/mL (n  =  3 at each level). The recovery of RR/SS-warfarin alcohols, WAR and RS/SR-warfarin alcohols was 60 ± 4%, 90 ± 3% and 70 ± 3%, respectively.

The inter- and intra-day precisions (%RSD) at a concentration level of 1000 ng/mL were 4% and 3% for RR/SS-warfarin alcohols, 3% and 4% for WAR, and 3% and 2% for RS/SR-warfarin alcohols, respectively.

The results showed that the total concentration of all the analytes (range, 200–2000 ng/mL) in human plasma samples was stable throughout the duration of a typical sequence of chromatographic analyses and for at least two months of storage at −80°C as well as after two cycles of thaw-freeze.

To determine the unbound plasma fraction of WAR and both diastereoisomers of WAROHs, an aliquot of plasma sample (1 mL) was centrifuged at 5000 rpm for 60 min at 25°C by an Amicon tube (Amicon Ultra-4, Millipore) with a molecular weight cut-off of 3 KDa. The filtrate sample (25 µL) was then injected into the HPLC-FLD system, without any further treatment.

The seven-point calibration curves (n  =  3 at each concentration) in the range 1 - 30 ng/mL were evaluated by the Deming regression analysis, and the best-fit models were (the slope values are reported with the corresponding standard deviation): y  =  (34200 ± 200) x, (R^2^ =  0.997) for RR/SS-warfarin alcohols, y  =  (12900 ± 100) x, (R^2^ =  0.999) for WAR and y  =  (20050 ± 100) x, (R^2^ =  0.996) for RS/SR-warfarin alcohols.

Plasma samples were spiked at 200, 1000 and 2000 ng/mL of WAR and both diastereoisomers of WAROHs (n  =  3 at each level) to obtain unbound plasma concentration levels at 2, 10 and 20 ng/mL, since the unbound plasma fraction is about 1% of the total content. These samples were used to determine inter- and intra-day precision, whereas the recovery was determined for standard working solutions at 2, 10 and 20 ng/mL (n  =  3 at each level).

The inter- and intra-day precisions (%RSD) at a concentration level of 10 ng/mL were 6% and 4% for RR/SS-warfarin alcohols, 8% and 5% for WAR, and 8% and 6% for RS/SR-warfarin alcohols, respectively. The recovery of RR/SS- and RS/SR-warfarin alcohols and WAR was 90 ± 8% and 70 ± 8%, respectively.

The unbound plasma fraction of all the analytes (range, 2–20 ng/mL) was stable throughout the duration of a typical sequence of chromatographic analyses and for at least two months of storage at 4°C. A 20% decrease was observed after the second freezing-thawing cycle at −80°C.

## Results and Discussion

### Octanol-Water partition coefficient (K_ow_) and dissociation constant of RR/SS- and RS/SR-warfarin alcohols

The value of LogK_ow_ at pH  =  7 of both diastereoisomers of WAROHs was determined by the standardized shake-flask method. Octanol was added to a working solution of the WAROHs prepared in PBS at pH 7.0. A working solution of WAR at 1000 ng/mL was also analyzed (n  =  3) to validate our method. The measured value of LogK_ow_ at pH  =  7 for WAR was 0.94 ± 0.05. This value is in good agreement with the one calculated by the equation reported in [29], which includes both pK_a_ and LogP (2.82 ± 0.06 at 25°C [15]) data. In fact, the difference between calculated and measured LogK_ow_ at pH  =  7 (Δ LogK_ow_  =  |LogK_ow_ _(calculated)_ – LogK_ow_ _(measured)_| was 0.02. The *t*-test showed that this difference was not statistically significant at a confidence level of 95%.

**Figure 2:**
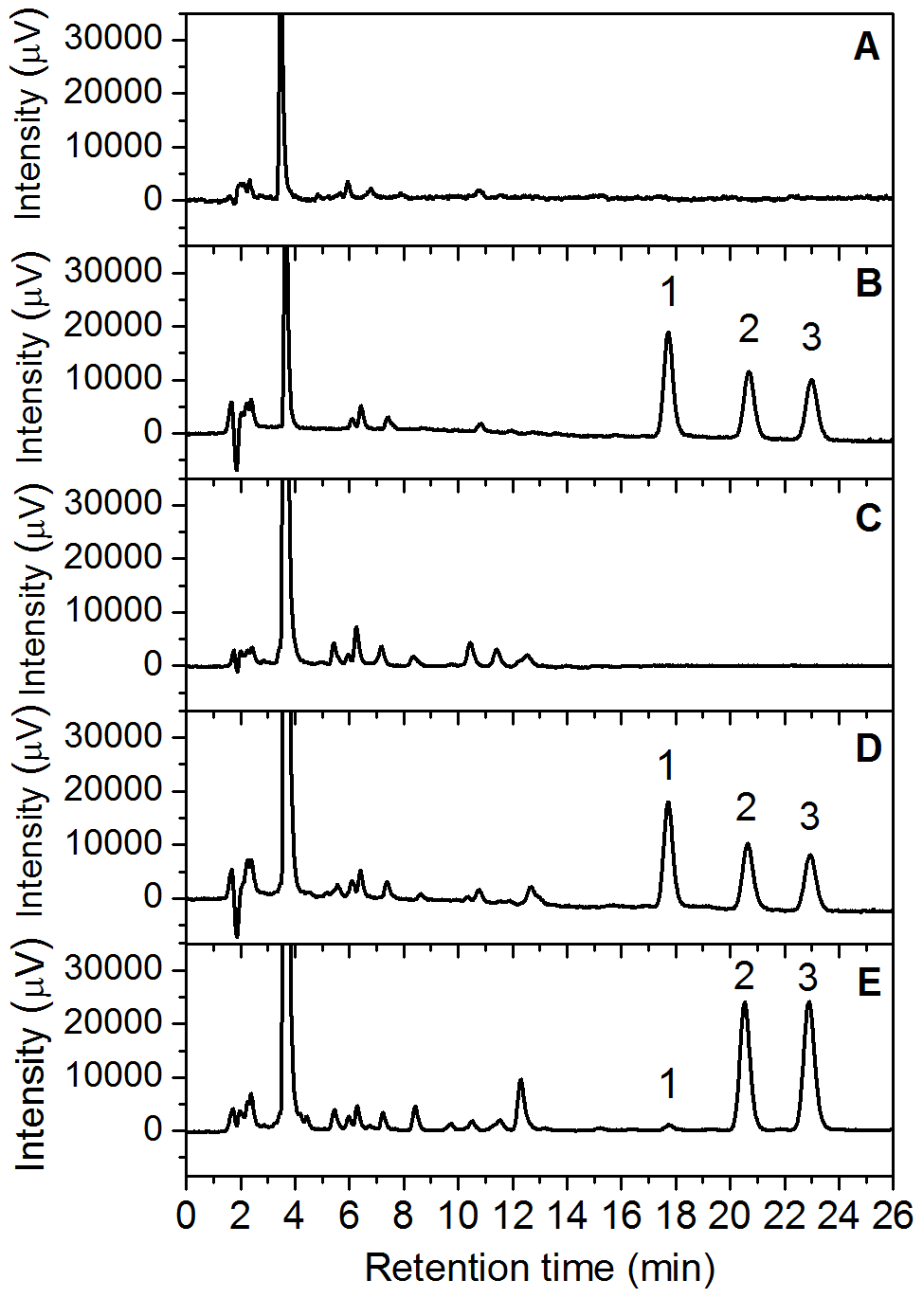
HPLC-FLD chromatograms of extracted samples: phosphate buffer solution at pH  =  7 (A), standard working solution of WAR and WAROHs (5 ng/mL) (B), control oral fluid samples (C), control oral fluid samples spiked with WAR and WAROHs (5 ng/mL) (D), and pooled patients oral fluid sample (0.6 ng/mL for RR/SS-warfarin alcohols, 9.8 ng/mL for WAR and 10.1 for RS/SR-warfarin alcohols) (E). Elution order and retention times: 1 (RR/SS-warfarin alcohols, r_t_: 17.5 min), 2 (warfarin, r_t_: 20.5 min), and 3 (RS/SR-warfarin alcohols, r_t_: 23.0 min).

The LogK_ow_ at pH  =  7 value for RR/SS- and RS/SR-warfarin alcohols were 0.83 ± 0.01 and 0.93 ± 0.01, respectively. The values were in good agreement with the peak elution order observed in our HPLC-FLD experimental conditions (**Figure 2**).

The dissociation constant (pK_a_) of both diastereoisomers of WAROHs was determined at different pH values of the mobile phase by the HPLC-FLD method by varying the organic solvent/water ratio. Thus the methanol/water solvent mixture is generally used because methanol shows a very similar solvation effect to water [30]. To validate our method a standard working solution of WAR at 1000 ng/mL was also analyzed (n  =  3). The measured pK_a_ value for WAR was 5.42 ± 0.03 at T  =  25°C, and was in good agreement with the literature [15]. The average difference Δ pK_a_ (Δ pK_a_  =  |pK_a__(literature)_ – pK_a__(measured)_| was 0.27. **Table 1** summarizes the p_s_K_a_ values of both diastereoisomers of WAROHs calculated using equation 1, at different percentages of methanol (R_wt%_).

**Table 1:** Cosolvent dissociation constant of RR/SS-warfarin alcohols and RS/SR-warfarin alcohols in different methanol-water mixtures.

R_wt%_	p_s_K_a_ (RSD a)	
	RR/SS-warfarin alcohols	RS/SR-warfarin alcohols
60	2.65 (0.4%)	3.01 (0.3%)
50	3.70 (0.3%)	3.68 (0.4%)
40	3.91 (0.5%)	3.89 (0.2%)

a Calculated from three standard working solutions of WAROHs.

The best aqueous pK_a_ values of RR/SS- and RS/SR-warfarin alcohols, obtained by plotting p_s_K_a_ *vs* R_wt%_ and extrapolating at zero organic content, were 6.55 ± 0.04, and 5.73 ± 0.03, respectively.

### Collection and pretreatment of oral fluid samples

#### Sample collection

Three sampling devices from Salivette (cotton swab, cotton swab impregnated with citric acid and synthetic swab) were compared to evaluate the recovery of WAR, RR/SS- and RS/SR-warfarin alcohols as well as the possible release of interfering compounds from the swab.

A standard working solution of WAR and WAROHs (5 ng/mL) was thus split into four sets with three aliquots of 2 mL each: the first was directly injected (25 µL) into HPLC-FLD system, whereas the other three were absorbed into the three different swabs and recovered by centrifugation at 3000 rpm for 5 min at room temperature. The centrifuged solutions were then injected (25 µL) into the HPLC-FLD system. The recovery was calculated from the ratio between the average concentrations of each analyte in the solution recovered from the swab and the initial concentration.

**Figure 3:**
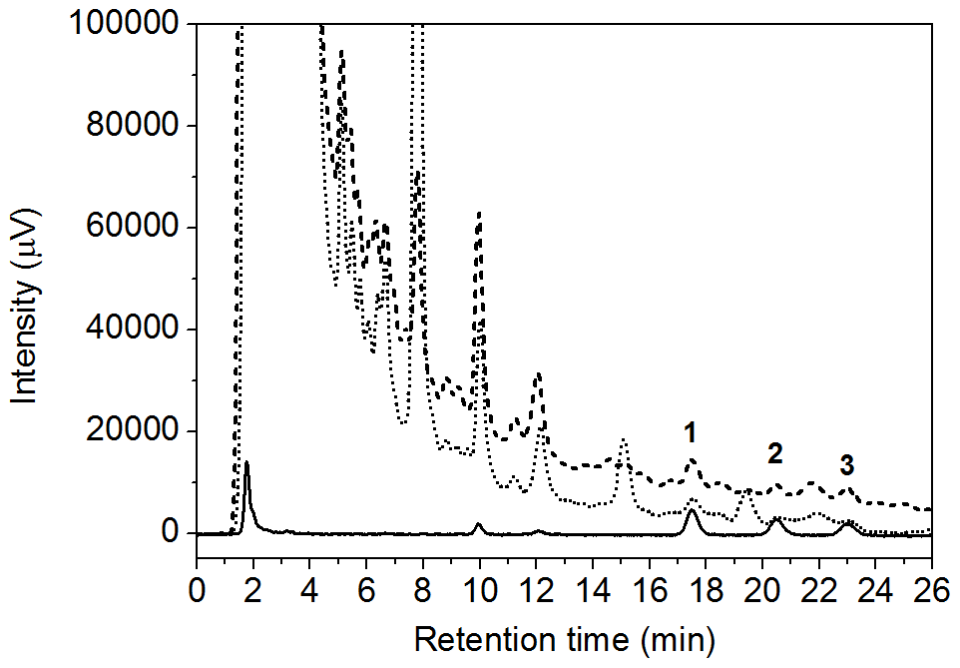
HPLC-FLD chromatograms of the WAR and WAROHs standard working solutions (5 ng/mL) recovered from different sampling devices: cotton swab (dashed line), cotton + citric acid swab (dotted line) and polyester swab (solid line). Elution order and retention times: 1 (RR/SS-warfarin alcohols, r_t_: 17.5 min), 2 (WAR, r_t_: 20.5 min), 3 (RS/SR-warfarin alcohols, r_t_: 23.0 min).

The results are summarized in **Figure 3** and **Table 2**, which highlight that the synthetic swab was the most suitable device for OF sampling because of the lowest background and the highest recovery percentage for WAR, RR/SS- and RS/SR-warfarin alcohols.

**Table 2:** Recovery of WAR, RR/SS- and RS/SR-warfarin alcohols in a standard working solution (5 ng/mL) from three sampling devices.

Swab	Average recovery % (RSD a)		
	RR/SS-warfarin alcohols	WAR	RS/SR-warfarin alcohols
Cotton	96% (5%)	88% (10%)	91% (6%)
Cotton + citric acid	75% (1%)	62% (3%)	76% (3%)
Polyester	100 (0.3%)	98% (1%)	98% (1%)

a Three replicates.

The recovery of both diastereoisomers of WAROHs from the synthetic swab was also estimated at four different pH values (5, 6, 7 and 8) of the OF samples. The corresponding recovery of WAR is described in [14].

A PSOFS at 5 ng/mL of WAROHs was split into three aliquots (6 mL): two aliquots were acidified at pH 5 and 6 by adding 7 and 5 µL of phosphoric acid 1 M respectively. The third aliquot was alkalinized to pH 8 by adding 8 µL of sodium hydroxide 1 M. Three aliquots (1 mL) of each of these samples were directly extracted with the procedure described in the Material and Methods, and three more aliquots (1 mL) were absorbed into the Salivette swabs. The solutions were recovered by centrifugation of the swabs at 3000 rpm for 5 min at room temperature and were then treated with the same extraction procedure. In this case too, the recovery was calculated from the ratio between the average RR/SS- and RS/SR-warfarin alcohol concentrations in the samples recovered from the synthetic swab and the initial concentration. A slight dependence on pH was found, as shown in **Table 3**.

**Table 3:** Recovery of RR/SS- and RS/SR-warfarin alcohols in a PSOFS (5 ng/mL) from synthetic swabs at pH values ranging from 5 to 8.

Measured pH	Average recovery % (RSD a)	
	RR/SS-warfarin alcohols	RS/SR-warfarin alcohols
5.1	94% (1%)	93% (5%)
6.1	97% (1%)	96% (3%)
6.9	100% (0.3%)	98% (0.3%)
8.2	99% (0.3%)	98% (0.3%)

a Three replicates.

Only a few patients showed a pH value of the non-stimulated OF lower than 6.0, thus the slight difference in the recovery percentage was considered negligible. In a few trials carried out with acidic OF samples, the small fraction of RR/SS- and RS/SR-warfarin alcohols remaining in the sampling swab was recovered by changing the sample pH to about 7.0 by adding 50–100 µL of PBS 1 M, reabsorbing the sample in the sampling swab and centrifuging the swab a second time. Recovery percentages were thus almost quantitative for both compounds.

The absorption of WAR and both diastereoisomers of WAROHs on the chewing gum used to stimulate the OF secretion was also examined. Standard working solutions of WAR and WAROHs (5 ng/mL) were added (n  =  3) to a piece of chewing gum, and vortex-mixed for 30 sec. After that, an aliquot (1 mL) of each mixture was then treated with the liquid-liquid extraction procedure. Neither statistically significant (P  =  1, two-tailed) differences between the concentrations in the treated samples compared to the initial concentration of standard working solutions, nor interferences in the HPLC-FLD chromatogram were observed (data not shown).

### Sample preparation

For the determination of WAR and RR/SS- and RS/SR-warfarin alcohols in OF we used the same procedure developed for the analysis of WAR and WAROHs in plasma samples [23] with small variations in the volumes used. The aim was to find the simplest and most reliable sample preparation procedure to remove the endogenous components (e.g. proteins, salts, lipids) that could interfere with the analyte determination. The recovery and precision for WAR and both diastereoisomers of WAROHs were determined during 5-day validation experiments. Three aliquots of a PCOFS spiked at different concentration levels (2, 10 and 20 ng/mL) were extracted in triplicate within the same day and on three consecutive days. **Table 4** reports the recovery of WAR, RR/SS- and RS/SR-warfarin alcohols as well as the corresponding intra- and inter-day relative standard deviations.

**Table 4:** Extraction recovery, intra- and inter-day precisions of the determination of WAR and RR/SS- and RS/SR-warfarin alcohols in spiked PCOFSs.

	Concentration (ng/mL)		Recovery %	Intra-day a RSD	Recovery %	Inter-day b RSD
	Expected	Measured				
RR/SS-warfarin alcohols	2.0	1.6	80	3%	81	3%
	10.3	9.2	89	4%	87	3%
	20.4	17.3	85	2%	86	4%
WAR	1.9	1.4	74	4%	73	5%
	9.5	7.3	77	1%	78	3%
	18.8	13.8	73	8%	75	2%
RS/SR-warfarin alcohols	2.0	1.7	85	2%	84	3%
	10.3	8.8	85	4%	87	4%
	20.4	17.5	86	3%	86	3%

a Three replicates.

b Three replicates.

### Interference and matrix effect

With the optimized mobile phase composition described above, a complete separation of all the analytes was achieved. The resolution factors (R_S_) for RR/SS-warfarin alcohols/WAR and WAR/RS/SR-warfarin alcohols were 4.3 and 3.0, respectively. **Figure 2** shows the HPLC-FLD chromatograms of the extracted alcohols: PBS at pH  =  7, standard working solution of WAR and WAROHs, COFS and COFS spiked with WAR and WAROHs and PPOFS.

In these experimental conditions, the retention times (t_r_) of RR/SS-warfarin alcohols, WAR and RS/SR-warfarin alcohols were 17.5, 20.5 and 23.0 min, respectively. The standard deviation of the retention time for the three analytes was always 0.1 min in ten replicate measurements.

The absence of interfering endogenous substances was confirmed by comparing the WAR, RR/SS- and RS/SR-warfarin alcohols concentrations of PSOFSs (5 ng/mL) and PPOFSs (unknown concentration) determined by the proposed method and by the HPLC-ESI-Q-TOF method described above. The concentration values obtained by the HPLC-ESI-Q-TOF and the HPLC-FLD, were in good agreement for all the samples analyzed.

For both methods, the slopes were not significantly different at a confidence level of 95%.

The absence of a matrix effect was also confirmed by comparing the slopes, reported with the corresponding standard deviation, of the calibration curves for a set of standard working solutions (RR/SS-warfarin alcohols: 30000 ± 1000, WAR: 13000 ± 700 and RS/SR-warfarin alcohols: 30000 ± 1000) and a set of extracted PPOFS samples (RR/SS-warfarin alcohols: 35400 ± 700, WAR: 14100 ± 600 and RS/SR-warfarin alcohols: 31800 ± 500). Again, the slopes were not significantly different at a confidence level of 95%.

### Calibration curves, limit of detection (LOD) and quantitation (LOQ)

A working range of 1–20 ng/mL was chosen for WAR, RR/SS- and RS/SR-warfarin alcohols. The five-point calibration curves (n  =  3 at each concentration) were evaluated by the Deming regression analysis, and the best-fit models were: y  =  (44000 ± 700) x, (R^2^ =  0.999) for RR/SS-warfarin alcohols, y  =  (19300 ± 600) x, (R^2^ =  0.999) for WAR and y  =  (30200 ± 400) x (R^2^ =  0.998) for RS/SR-warfarin alcohols.

A blank OF sample spiked with 0.5 ng/mL of WAR and WAROHs was treated five times with the liquid-liquid extraction procedure and then analyzed by HPLC-FLD.

The LOD and LOQ values were calculated in accordance with IUPAC guidelines [24], as three and ten times the standard deviation (sb) of the “low level spiked blank”, and were 0.1 and 0.3 ng/mL for RR/SS-warfarin alcohols, 0.2 and 0.6 ng/mL for WAR and 0.2 and 0.5 ng/mL for RS/SR-warfarin alcohols.

### Sample stability

The stability of the standard working solutions of WAR and both WAROHs diastereoisomers and OF samples was evaluated in triplicate following the experimental plan reported in **Table 5**. The temperature and duration of storage were chosen in order to reflect the typical conditions of clinical studies. The concentrations of each analyte at t  =  0 h were used as the reference value. The stability of the samples was evaluated by an ANOVA test at a confidence level of 95%.

**Table 5:** Experimental plan of time stability studies for WAR, RR/SS- and RS/SR-warfarin alcohols.

	Concentration (ng/mL)	Storage conditions		
		2 months	24 h	Freeze-thaw cycle
Standard working solution	2, 10, 20	4°C	RT a	—
Pooled patients oral fluid sample	2, 10, 20	−80°C	RT	2 cycles
Extracted oral fluid sample	10	4°C	RT	—

a Room Temperature.

The results showed that the concentrations of all the analytes in the OF samples were stable at −80°C (two cycles of freezing-thawing) over a period of two months. In addition, no significant degradation occurred in the extracted OF samples and standard working solutions throughout the duration of a typical sequence of chromatographic analyses (storage in the autosampler for about 24 h at room temperature) and for at least two months of storage at 4°C.

### Influence of the collection method on the oral fluid concentration of warfarin, RR/SS- and RS/SR-warfarin alcohols

The analytical method was applied to the determination of WAR, RR/SS- and RS/SR-warfarin alcohols in OF samples collected from 14 patients undergoing WAR therapy. In this work, three different sampling protocols (see Material and Methods) were evaluated to: (i) establish the equilibrium time between blood and OF for all the analytes under OF stimulation, (ii) optimize the stimulated OF sampling, and (iii) determine the repeatability of the stimulated sampling procedure.

**Figure 4:**
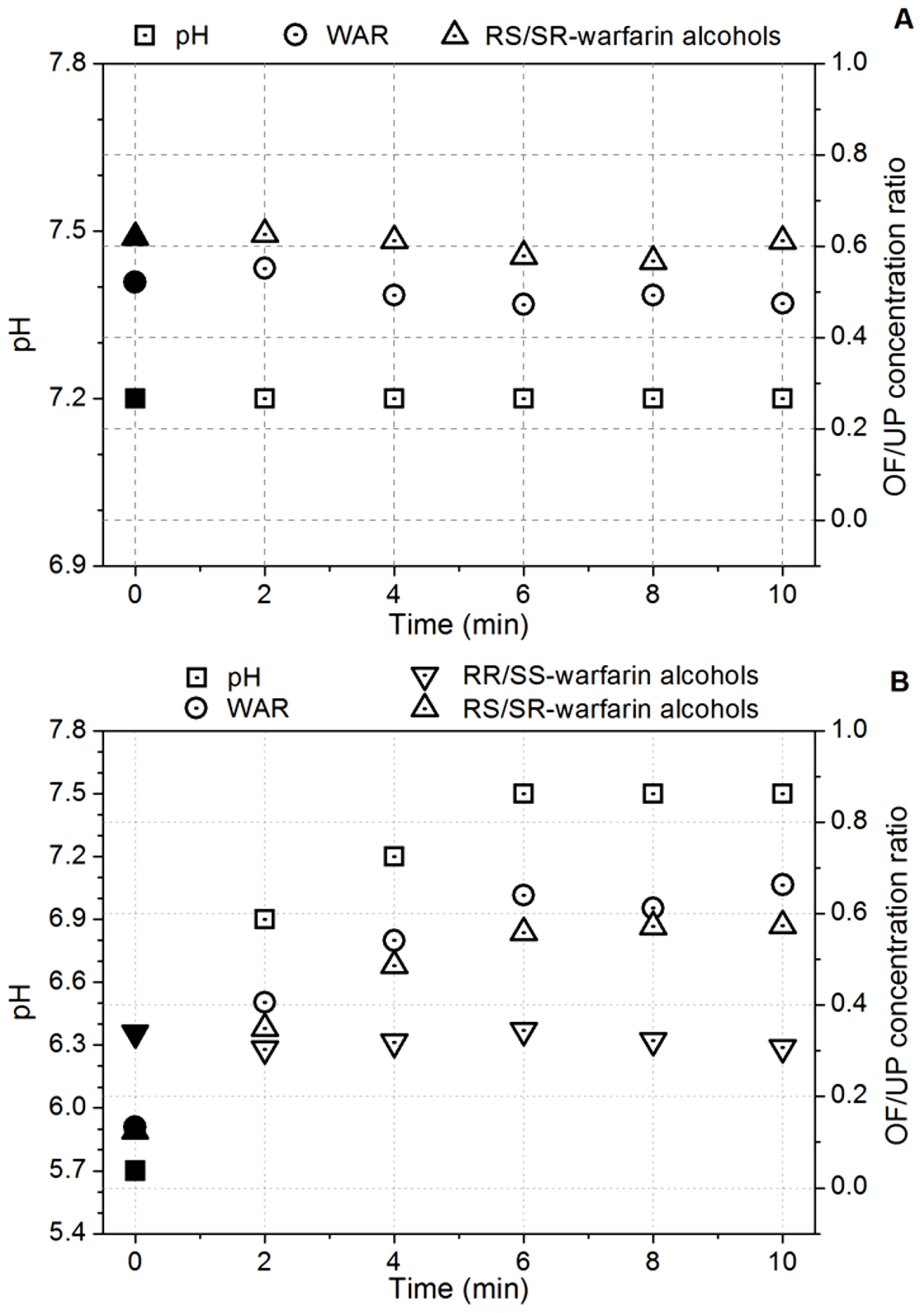
Effect of oral fluid pH on the oral fluid/unbound plasma concentration ratio of WAR, RR/SS- and RS/SR-warfarin alcohols for patients P5 (A), P6 (B) undergoing warfarin therapy.

**Figure 4** shows the effect of sampling time of OF stimulation on both pH as well as the oral fluid/unbound plasma concentration ratio (OF/UP) of WAR, RR/SS- and RS/SR-warfarin alcohols for patients P5 and P6. The values reported at t  =  0 min corresponded to non-stimulated OF samples. The non-stimulated OF samples were obtained according to procedure 1, whereas the stimulated OF was collected by procedure 2 at five different times (t  =  2, t  =  4, t  =  6, t  =  8 and t  =  10 min).

The OF/UP concentration ratio of WAR, RR/SS- and RS/SR-warfarin alcohols was evaluated in relation to the concomitant pH variation.

The distribution of a drug at the side of the salivary membrane is generally determined by its pK_a_ and LogK_ow_ values as well as by the pH values in plasma and in oral fluid [16]. A variation in OF pH (pH in plasma is buffered at 7.4) influences the concentrations of the drug, since, at equilibrium the non-ionized form, which is the only one able to diffuse across the membrane [31], will have the same concentration at each side of the membrane. Acidic drugs (such as WAR and RS/SR-warfarin alcohols), which are largely dissociated in plasma, show an increase in the OF/UP concentration ratio with an increase in salivary pH, whereas the OF/UP concentration ratio of neutral drugs (e.g. RR/SS-warfarin alcohols) is not influenced by the pH.

**Figure 4A** shows the typical situation of about one third of the enrolled patients with a constant salivary pH value close to that of blood. No difference between non-stimulated (t  =  0 min, full symbol) and stimulated (t ≠ 0 min, empty symbol) OF/UP concentration ratios of WAR and RS/SR-warfarin alcohols were observed in patient P5 as a consequence of the similar pH values on both sides of the salivary membrane.

**Figure 4B** shows what happened when a marked increase in salivary pH was observed in the remaining patients. The change in salivary pH from 5.7 to 7.5 observed in patient P6 during stimulation was associated with a corresponding synchronous increase in both WAR and RS/SR-warfarin alcohols concentrations. On the other hand, RR/SS-warfarin alcohols remained practically constant throughout the whole sampling period. RR/SS-warfarin has an almost neutral pK_a_ value (6.55 ± 0.04), so that its concentration in OF is hardly influenced by pH.

These results also provide useful information on the kinetics of the diffusion process controlling the concentrations of WAR and RS/SR-warfarin alcohols in OF. **Figure 4B** shows that pH reaches equilibrium under stimulation in about 6 minutes, and that WAR and RS/SR-warfarin alcohols immediately follow pH variations. As pH is the variable controlling the diffusion of WAR and RS/SR-warfarin alcohols, these results suggest that OF sampling should be performed at least after 6 minutes and that the diffusion equilibrium of the drug at a certain pH value is established in less than 2 minutes (i.e. the time the swab was kept in the mouth).

With regard to these results, we investigated the repeatability of the proposed stimulated sampling procedure for all the patients enrolled. The sampling procedure comprised two different steps: a) chewing of sugar-free chewing gum for 6 min, and b) the consecutive collection of five different stimulated OF samples. The five OF samples were obtained by rolling a synthetic swab over a period of 2 min, during which the stimulation was maintained as constant as possible. We found that stimulated OF samples could be collected and had an RSD lower than 10% for the measured concentrations of WAR and both diastereoisomers of WAROHs, although an expected inter-individual variability was observed.

### Correlation between warfarin, RR/SS- and RS/SR-warfarin alcohols concentration in oral fluid and plasma samples

Finally, the sampling procedures outlined above were used to collect non-stimulated and stimulated OF samples from 14 patients undergoing WAR therapy. The aim was to evaluate the correlations between the OF and plasma (unbound and total) concentrations of WAR, RR/SS- and RS/SR-warfarin alcohols.

**Figure 5:**
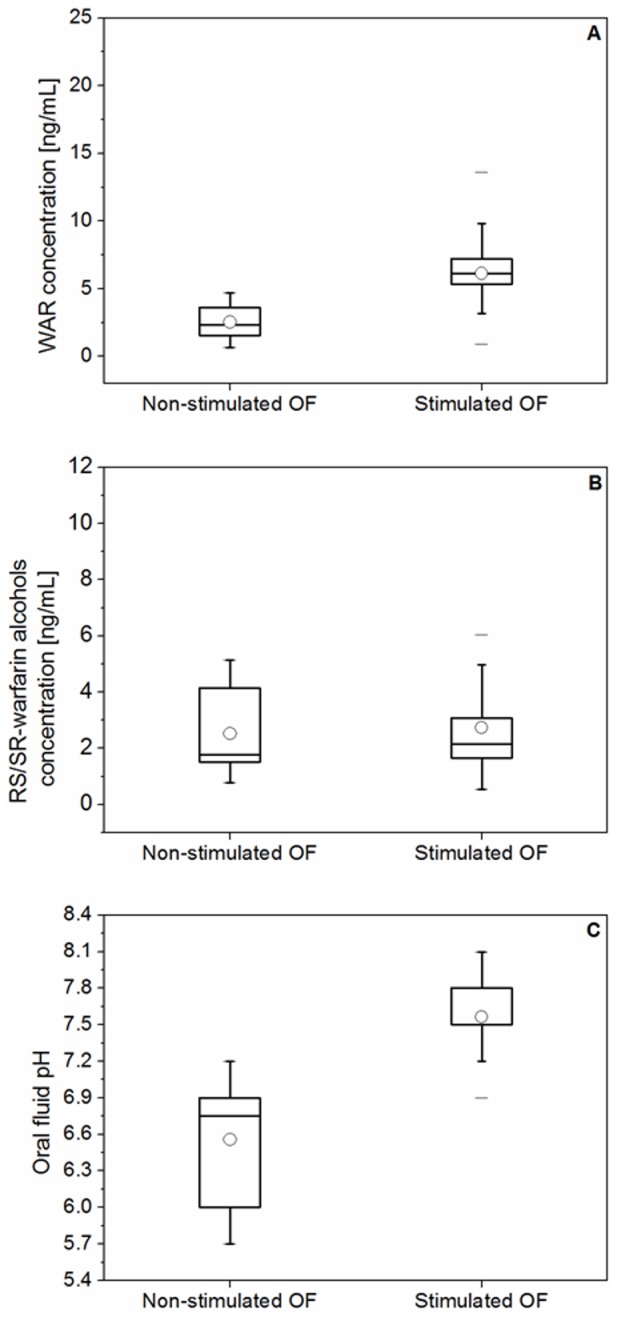
Box-plot for warfarin concentration (A), RS/SR-warfarin alcohols concentration (B) and pH (C) for non-stimulated and stimulated OF samples from 14 patients undergoing warfarin therapy. Note: The box-plot shows: the minimum, the 5^th^ and the 25^th^ percentiles, the median, the 75^th^ and 95^th^ percentiles, and the maximum value for each variable investigated. The dot inside the box shows the mean value.

The concentration (mean ± s.d.) of WAR (**Figure 5A**) and RS/SR-warfarin alcohols (**Figure 5B**) in the non-stimulated and stimulated OF samples were 3 ± 2 ng/mL (range, 1–5 ng/mL) and 3 ± 2 ng/mL (range, 1–5 ng/mL), and 6 ± 3 ng/mL (range, 1–14 ng/mL) and 3 ± 2 ng/mL (range, 1–6 ng/mL), respectively. Stimulation increased pH values (mean ± s.d.) from 6.6 ± 0.4 (range, 5.7–7.2) to 7.5 ± 0.3 (range, 6.9–8.1) (**Figure 5C**). The OF flow rate values (mean ± s.d.) increased from 0.10 ± 0.05 mL/min (range, 0.05–0.20 mL/min) up to a much higher value, which then caused the oversaturation of the swab during the sampling time (2 min).

Unbound plasma concentrations (mean ± s.d.) of WAR and RS/SR-warfarin alcohols were 9 ± 4 (range, 3–17 ng/mL) and 5 ± 3 ng/mL (range, 1–10 ng/mL), respectively. Only four patients showed concentrations of RR/SS-warfarin alcohols in OF (both in non-stimulated and stimulated samples) and plasma (unbound fraction) higher than the LOQ value.

Total plasma concentrations (mean ± s.d.) of RR/SS-warfarin alcohols, WAR and RS/SR-warfarin alcohols were 30 ± 20 (range, 13–82 ng/mL), 1300 ± 500 (range, 400–2000 ng/mL) and 600 ± 300 (range, 100–1100 ng/mL), respectively.

The Mann-Whitney test did not highlight statistically significant gender differences (p <0.05) for any of the above parameters.

**Figure 6:**
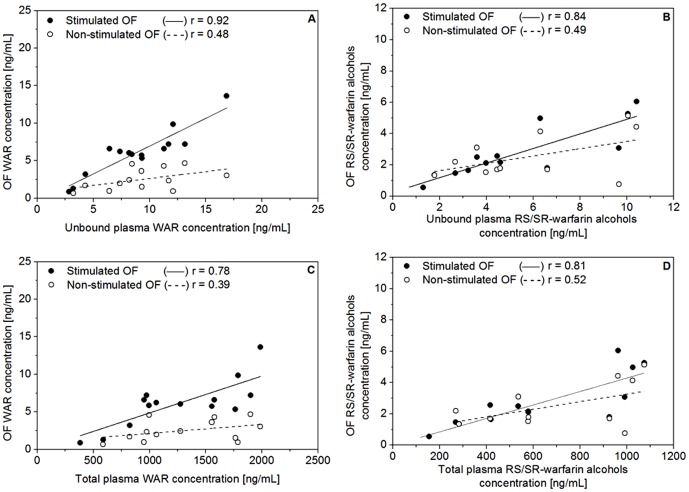
Concentrations of warfarin (A and C), and RS/SR-warfarin alcohols (B and D), in non-stimulated and stimulated oral fluid versus unbound and total plasma concentrations in 14 patients undergoing warfarin therapy.

**Figure 6** shows the relationship between the concentration of WAR and RS/SR-warfarin alcohols in OF and plasma samples. The relationship between the variables was examined by Deming's linear regression (C_OF_  =  S×C_i_ ± C_0_ where C_OF_ is the OF concentration, S is the slope, C_i_ is either C_UP_ (unbound plasma concentration) or C_P_ (total plasma concentration), and C_0_ is the intercept.

A strong correlation was observed between the stimulated OF and the unbound plasma concentration of WAR (**Figure 6A**, r  =  0.92, p <0.001, C_OF_  =  0.740×C_UP_ – 0.450) and RS/SR-warfarin alcohols (**Figure 6B**, r  =  0.84, p <0.001, C_OF_  =  0.450×C_UP_ + 0.340) as well as between stimulated OF and total plasma concentration of WAR (**Figure 6C**, r  =  0.78, p <0.001, C_OF_  =  0.005×C_P_ – 0.070) and RS/SR-warfarin alcohols (**Figure 6D**, r  =  0.81, p <0.001, C_OF_  =  0.004×C_P_ + 0.090).

These results confirmed the hypothesis of the key-role of salivary pH in controlling the membrane transfer of both analytes from blood to OF. If the diffusion mechanism is passive and fast, the stimulated OF concentration should be close to the concentration of the unbound plasma fraction since OF and plasma pH values are very similar. In fact, the stimulated OF/UP ratio (mean ± s.d.) of WAR and RS/SR-warfarin alcohols was 0.7 ± 0.2 (range, 0.3–0.9) and 0.5 ± 0.2 (range, 0.3–0.8), respectively. The non-stimulated OF/UP ratio (mean ± s.d.) was 0.3 ± 0.1 (range, 0.1–0.5) and 0.4 ± 0.3 (range, 0.1–0.8), respectively.

These results also highlight that the stimulated OF sampling method reduced the inter-subject variability (%RSD) of the OF/UP ratio from 60% to 30%. This experimental evidence is a consequence of the lower pH difference between OF and plasma compared to the non-stimulated sampling method.

Since adsorption of WAR and RS/SR-warfarin alcohols on the surface of both the chewing gum and synthetic swab was ruled out by the quantitative recovery of analytes, an OF/UP concentration ratio of WAR and RS/SR-warfarin alcohols lower than 1 may be related to variations in the OF flow rate.

Higashi *et al* reported that for lipophilic compounds (Log K_ow_ values at pH  =  7 higher than 1.76) the major route for entry into OF is a rapid diffusion through the acinar cells (the transcellular route). Therefore, the rate of diffusion across the cells is so fast that the OF concentrations are independent of the rate of salivary secretion. In contrast, compounds with limited lipid solubility (Log K_ow_ values at pH  =  7 lower than 0.02) may principally enter the saliva via the tight junctions of the acinar cells (the paracellular route) at a low rate, and the concentrations are significantly influenced by the OF flow rate [16], [20], [32], [33].

In the case of WAR and RS/SR-warfarin alcohols, Log K_ow_ values at pH  =  7 are in the range 0.83–0.94, which places the analytes in an intermediate zone compared to cases reported by Higashi *et al*. Although the OF flow rate may have influenced the measured concentration ratio of WAR and RS/SR-warfarin alcohols, it was not possible to quantitatively estimate this possible effect. This was because during stimulated OF sampling, the swab was often oversaturated which prevents any quantitative assessment of OF the flow rate.

However, this aspect is not crucial for WAR therapy monitoring, because the OF/UP concentration ratio should be constant for each patient if the intensity of stimulation is kept constant throughout the whole collection period [19].

The simultaneous measurement of UP and total plasma determines the drug binding to proteins.

The protein-binding (mean ± s.d.) of WAR, RR/SS- and RS/SR-warfarin alcohols was 99.2 ± 0.3%, 98.8 ± 0.4% and 99.3 ± 0.2%, respectively. These results are comparable to 99.1% for WAR obtained from 86 patients using 14 C-labeled WAR and ultrafiltration with Centrifree devices [34]. No data on the protein binding to albumin of both diastereoisomers of WAROHs are available in the literature. The value calculated (about 99%), which was close to that reported for WAR, is not surprising because both analytes have the same chemical structure (e.g. coumarin) and probably essentially bind, in the same way, to Sudlow's site I of albumin.

In conclusion, we optimized the OF sampling procedure including testing various collection devices and sampling methods (i.e. non stimulated and stimulated). A six-minute stimulation with chewing gum followed by the collection with a polyester swab was the best sampling procedure, with an optimum repeatability (RSD <10%). In fact, the synthetic swab combined the highest recovery and the lowest blank level. The stimulated OF sampling also led to an increase in the OF pH, which approached the physiological value in blood (7.4) in about 6 minutes. This stimulated OF sampling procedure also reduced the inter-subject variability (%RSD) of the OF/UP ratio from 60% to 30% compared to the non-stimulated procedure.

In the optimized conditions, the concentration of WAR and RS/SR-warfarin alcohols in OF increased with the pH value, whereas the concentration of RR/SS-warfarin alcohols was not affected.

When salivary pH was close to blood pH, strong correlations between the concentrations of both WAR and RS/SR-warfarin alcohols in the stimulated OF and the unbound plasma fraction were observed.

These results indicate that OF analysis has the potential to become an efficient clinical tool for daily non-invasive therapy monitoring.
